# The potential impact of COVID-19 disease caused multi-organ injuries on patients' surgical outcomes

**DOI:** 10.1007/s44254-023-00004-8

**Published:** 2023-03-10

**Authors:** Sanketh Rampes, Daqing Ma

**Affiliations:** Division of Anaesthetics, Pain Medicine and Intensive Care, Department of Surgery and Cancer, Faculty of Medicine, Imperial College London, Chelsea and Westminster Hospital, London, SW10 9NH UK

**Keywords:** COVID-19, Long-covid, Surgery, Anasthetics, Perioperative

## Abstract

**Purpose:**

To provide an expert commentary on the impact of prior COVID-19 infection on patient’s surgical outcomes and postoperative recovery. To highlight the need for greater focus on peri-operative care of patients who have recovered from COVID-19.

**Methods:**

A narrative review of the literature was conducted by searching Pubmed and EMBASE for relevant articles using keywords such as “COVID-19”, “Coronavirus”, “surgery” and “peri-operative infection”.

**Results:**

Post-COVID-19 condition also known as long COVID has an estimated incidence of between 3.0 to 11.7%. COVID-19 has been shown to cause a series of short and long-term sequelae including cardiopulmonary complications, renal impairment, chronic fatigue and muscular deconditioning. Peri-operative infection with COVID-19 is associated with increased peri-operative mortality. Elective surgery patients who developed COVID-19 were 26 times more likely to die whilst in hospital compared to controls without COVID-19 infection, and for emergency surgery patients with COVID-19 infection were six times more likely to die. A large international prospective cohort study identified that patients who had surgery delayed over 7 weeks from the date of COVID-19 infection had no increased 30-day postoperative mortality, except those with ongoing symptoms.

**Conclusions:**

COVID-19 infection and its complications have been shown to adversely affect surgical outcomes. Further research is required to better characterise long COVID and the long-term sequelae that develop, which should be used to guide comprehensive peri-operative assessment of patients.

**Graphical Abstract:**

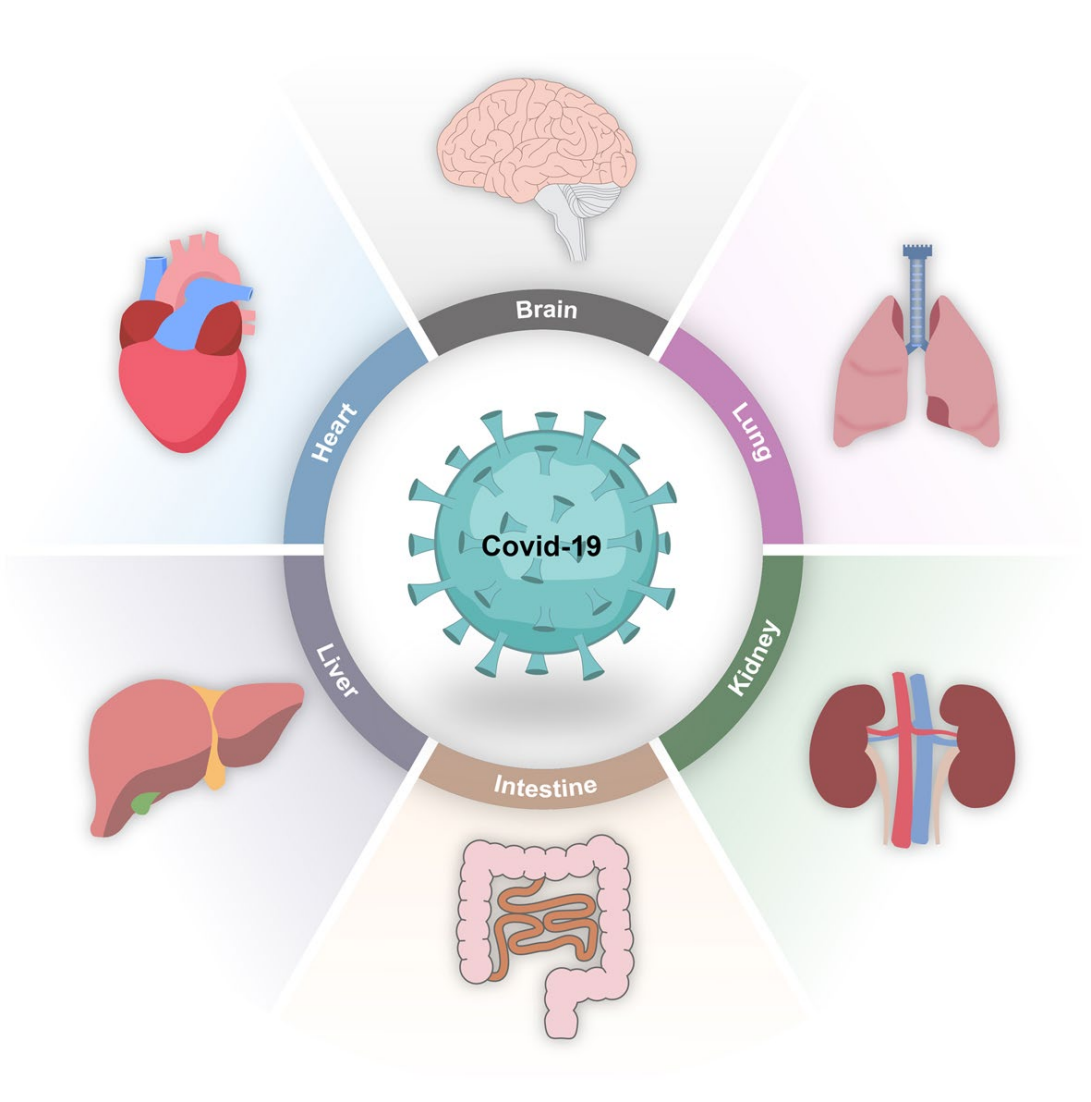

Coronavirus disease 2019 (COVID-19) is an infectious disease caused by SARS-CoV-2 and as of 21^st^ October 2022, there were 624 million infections and 6.55 million deaths globally [1]. Although the virus primarily causes a respiratory infection, COVID-19 affects multiple systems [2] and has both short and long-term sequelae including lung damage, cardiac injury, renal impairment, neurological complications, thromboembolic complications, chronic fatigue and musculoskeletal deconditioning [3]. These complications of COVID-19 may impact on patients’ surgical outcomes and postoperative recovery and therefore should be considered when planning elective surgery. The editorial highlights the need to focus greater attention on peri-operative care of patients who have recovered from COVID-19.

Post-COVID-19 condition also known as long COVID has recently been defined by the WHO Delphi consensus of “Post-COVID-19 condition occurs in individuals with a history of probable or confirmed SARS-CoV-2 infection, usually 3 months from the onset of COVID-19 with symptoms that last for at least 2 months and cannot be explained by an alternative diagnosis. Symptoms might be new onset after initial recovery from an acute COVID-19 episode or persist from the initial illness. Symptoms might also fluctuate or relapse over time” [4]. Although long COVID was identified as a phenomenon early into the pandemic compared to acute infection, the epidemiology, risk factors and management are poorly understood.

In the UK, the Office for National Statistics (ONS) estimates the incidence of post-COVID-19 condition 12 weeks following COVID-19 infection to vary from 3.0% based on tracking specific symptoms to 11.7% based on self-classification of long COVID [5]. Studies suggest that long COVID tends to be more prevalent in females, and the risk of persistent symptoms is linearly related to age [6, 7]. The severity of long COVID appears to be independent of the severity of acute infection with COVID-19, and a study of low-risk patients with persistent symptoms found impairment of at least one organ four months after initial infection even in non-hospitalised patients [8]. There are currently nine ongoing large national studies within the UK to better characterise and understand long COVID [9]. Multiple studies (CONVALESNCE, CLoCk, TLC and REACT) employ control groups that are matched for age and co-morbidities and are particularly enlightening in prevalence of symptoms specific to long COVID [10].

Peri-operative infection with COVID-19, including patients who experienced post-COVID-19 condition, is associated with an increased post-operative mortality [11–13]. Elective surgery patients who developed COVID-19 were 26 times more likely to die while in hospital compared to those without COVID-19 infection, and for emergency surgery patients those who had COVID-19 infection were six times more likely to die when in hospital [11]. A recent international prospective cohort study aimed to identify the optimum timing of delay in patients who had COVID-19; surgical patients with preoperative COVID-19 were compared to patients without preoperative COVID-19 and the outcomes of interest was 30-day postoperative mortality [14]. With the adjusted logistic regression analysis, it was found that patients with a pre-operative diagnosis of COVID-19 had a higher odds ratio of mortality having surgery within 0–2 weeks (4.1; 95%CI 3.3–4.8), 3–4 weeks (3.9; 95%CI 2.6–5.1) and 5–6 weeks (3.6; 95%CI 2.0–5.2) of the diagnosis [14]. In patients having surgery who had a diagnosis of COVID-19 > 7 weeks prior to surgery, there was no increased 30-day postoperative mortality. However, amongst those, patients having ongoing symptoms beyond 7 weeks had a higher post-operative mortality (6.0%; 95%CI 3.2–8.7) compared with those whose symptoms had resolved (2.4%; 95%CI 1.4–3.4), or were asymptomatic (1.3%; 95%CI 0.6–2.0) [14]. A similar pattern was seen with postoperative pulmonary complications, whereby patients who were at a greater risk of postoperative pulmonary complications up to 6 weeks after COVID-19; however, at 7 weeks the rates were similar when compared with those without COVID-19 [14]. Other studies have also reported similar patterns during postoperative period [15]. In propensity-matched analysis, septic and pulmonary complications were increased beyond 30 days of a positive test, and the rate of ischaemic stroke was increased up to 30 days after a positive test [15]. Studies also suggest that patients with resolved symptoms were at greater risk of 30-day mortality when compared to those with asymptomatic disease. This suggests that the current and past clinical status of patients are important when considering the timing of surgery [16].

Patients with COVID-19 having had critical care admission may require special consideration as not only will these patients have increased severity of pathological sequalae of COVID-19, they will also be deconditioned and require physical rehabilitation. Additionally, the use of immunosuppressive therapies in patients with COVID-19 including toclizumab and sarilumab may lead to increased risk of postoperative infection and delayed wound healing [17], and, therefore, guidance from our rheumatology colleagues may be warranted about the timing of surgery.

COVID-19 has been shown to cause a series of short and long-term sequelae including cardiopulmonary complications, renal impairment, chronic fatigue and musculoskeletal deconditioning [3, 18–20]. Further research is required to better characterise long-COVID and the long-term sequelae that develop following acute COVID-19 infection. The ongoing studies discussed above will be informative in answering these questions. Studies conclusively show that postoperative mortality for both elective and emergency surgery is increased following COVID-19 infection, and the optimal duration for postponing surgery following infection seems to be 7 weeks, except in those with ongoing symptoms. Studies also showed that both current and previous clinical status when considering COVID-19 infection is relevant to postoperative outcomes [16]. Comprehensive perioperative assessment of patients must be conducted to include co-morbidities, clinical course of COVID-19 infection, any current ongoing symptoms of long-COVID and a comprehensive organ-based assessment. These factors should all be taken into close consideration when considering surgery in patients recovering from COVID-19. A multidisciplinary consensus statement on behalf of several professional medical organisations in the UK made a series of recommendations [16], many of which have been discussed in this commentary; however as further evidence emerges it is likely that the recommendations will become more extensive and robust.

## Data Availability

N/a.
